# Quality of life of patients with neurofibromatosis 1—Physical disability does not necessarily result in poor mental health

**DOI:** 10.3389/fneur.2024.1432196

**Published:** 2024-10-30

**Authors:** Ute Bäzner, Leonie Stauss, Thomas Kapapa, Christian Rainer Wirtz, Andrej Pala

**Affiliations:** ^1^Department of Neurosurgery, Bezirkskrankenhaus Günzburg, University of Ulm, Günzburg, Germany; ^2^Department of Neurosurgery, University of Ulm, Universitätsklinikum Ulm, Ulm, Germany

**Keywords:** NF1, quality of life, SF-36, severity, visibility

## Abstract

**Introduction:**

Neurofibromatosis 1 (NF1) is a chronic neurocutaneous disease known to profoundly affect quality of life (QoL). We have performed an analysis of disease severity, mental and physical QoL and compare the different subclasses among patients with neurofibromatosis 1 (NF1).

**Patients and methods:**

We conducted a prospective analysis of 89 NF1 patients between January 2016 and March 2018. Data sourced from local records including demographic information, employment status, education level, and marital status. All patients completed 36-Item Short Form Health Survey (SF-36) and additionally the numerical pain rating scale (NPS). Patients were stratified based on severity of NF1, visibility and disease severity.

**Results:**

Among 89 patients, severity was classified as grade 4 was identified in 42 (47.2%), moderate in 17 (19.1%), mild in 23 (25.8%) and minimal in 7 (7.9%) cases. According to visibility scale, severe grade 3 was found in 28 (31.5%), moderate grade 2 in 26 (29.2%) and mild grade in 35 (39.3%) cases. SF-36 data, except for pain, showed significantly lower values, if compared to the standard German population (*P* < 0.001, physical component summary *P* = 0.045). Sex, marital status and education level did not significantly influence results. Employment was significantly associated with better mental and physical status (*P* = 0.028 and *P* = 0.01 respectively) and age >40 was linked to lower physical (*P* = 0.027) but not mental component scores (*P* = 0.362). The numerical pain rating scale indicated pain levels of 7–10 in 9 cases (10,1%), 5–6 in 10 patients (11.2%), 1–4 in 26 patients (29.2%) and no pain in 44 cases (49.4%). Physical component scores significantly differed across different NPS grades (*P* < 0.001) but not in mental component scores (P = 0.06). Finally, no significant differences were found in mental component scores across severity or visibility grades.

**Conclusion:**

Severity and visibility grades of patients with NF1 may not necessarily result in poor mental health. Symptomatic treatment should be considered even for severely disabled patients as they may have comparable QoL to less severely affected patients with NF1. Employment was linked to better quality of life outcomes in our findings.

## Introduction

Neurofibromatosis is a heterogeneous neuro-cutaneous disease, including neurofibromatosis type 1 (NF1), neurofibromatosis type 2 (NF2) and schwannomatosis (1, 2). NF1, the most common subtype (1:2,700 births), is characterized by many typical features such as café-au-lait macules (CALM), freckling, Lisch nodules, cutaneous and plexiform neurofibromas, optic gliomas, bone deformities and in addition associated learning difficulties and attention deficits (ADHD) with different impact on quality of life (QoL) (1–3). Plexiform neurofibromas pose the risk not only for malignant transformation resulting in malignant peripheral nerve sheath tumors (MPNST) but also for visible deformities which may influence QoL (4). NF1 patients have an increased risk of developing several other tumor diseases like gastrointestinal or breast cancers, impacting the life expectancy (5, 6). These patients often undergo repetitive surgeries and develop different neurological impairments due to tumor progression, so that decision making for an aggressive treatment is difficult and the severity grade may influence the final choice.

Health related Quality of Life (QoL) encompasses physical, psychological and social wellbeing of patients and reflecting the complex and multifactorial conditions and factors affecting the patients‘ lives (7). The aim of this study is to identify the various aspects of QoL in patients with NF1 and to identify predictors of QoL within this population. Despite previous studies described an association between neurofibromatosis and a diminished QoL, our knowledge about the factors which influence the QoL of this group is limited (1, 3). Furthermore, we have analyzed the impact of employment, age, marital status and education level on QoL.

## Patients and methods

### Study design

We conducted a prospective, descriptive, observational study between 2016 and 2018 involving 89 patients. The patients were consecutively enrolled in the study. The study received approval from the local ethics committee (N 51/16) and adhered to the principles of the international Declaration of Helsinki. Informed consent was obtained from all participants (3, 8).

### Collected data

A standardized, generic survey for health-related quality of life was chosen to ensure reproducibility and comparability in this topic. All patients attending the specialized NF outpatient department and providing informed consent were included in the study. The advantage of local NF outpatient department lies in interdisciplinary approach involving neurologists, neurosurgeons, dermatologists, pediatricians and genetics experts. Each patient underwent a neurological examination and received the 36-Item Short Form Health Survey (SF-36) and numerical pain rating scale (9, 10). The local records were analyzed for demographic data and patients were stratified based on severity and visibility grades as proposed by Huson et al. and Ablon et al. (3, 8).

Demographic variables including age, sex, marital status and employment status were analyzed. Two age groups with cut of >40 years were evaluated. The cut off value was based on assumption that it is the middle of life episode, typically with grounded family.

### Statistical analysis

Descriptive statistics were used for data analysis, reporting mean and standard deviation for continuous variables and absolute and relative frequencies for qualitative parameters. Explorative tests between interesting subgroups were applied by the underlying parameters (ANOVA as well as T-test). Non-parametric tests were performed in the presence of non-Gaussian distribution of values (Mann-Whitney Test, Kruskal-Vallis-test). The significance level was defined as *P* < 0.05. Statistical analysis was performed using the SPSS^®^ statistical software (IBM Company, SPSS Inc. Chicago Illinois).

## Results

### Demographic and general data

Detailed demographic data including severity and visibility scores and other NF1 characteristics are summarized in Table 1. Females predominated in our cohort and the majority of patients were employed. Slightly more patients are married or are living in a relationship. Approximately, half of the patients have a familial form of NF1 (Table 1).

**Table 1:** Patients'characteristics.

**Sex**	**Male**	***N =* 33 (37.1%)**	***P* = 0.015**
	Female	*N* = 56 (62.9%)	
Age	Mean (SD)	38.67 ± 16.12	
Inheritance	Familial	42 (47.2%)	*P* = 0.596
	Sporadic	47 (52.8%)	
Partner/Marital status	Single	42 (47.2%)	*P* = 0.596
	Partner/married	47 (52.8%)	
Level of education	None	9 (10.1%)	*P* < 0.001
	Elementary school	36 (40.4%)	
	Middle school	24 (27%)	
	High school	20 (22.5%)	
Employment	Employed	76 (78.4%)	*P* < 0.001
	Unemployed	13 (21.6%)	
Disease severity	Minimal (Grade 1)	7 (7.9%)	*P* < 0.001
	Mild (Grade 2)	23 (25.8%)	
	Moderate (Grade 3)	17 (19.1%)	
	Severe (Grade 4)	42 (47.2%)	
Visibility scale	Mild (Grade 1)	35 (39.3%)	*P* = 0.471
	Moderate (Grade 2)	26 (29.2%)	
	Severe (Grade 3)	28 (31.5%)	

Regarding education level, it's visible that 10.1% of the patients are without any graduation, which is more than twice as much as in the general German population (4.7%).

Severe grades were observed in nearly half of the patients (*N* = 42, 47.1%) in our study, typically correlating with increased surgeries and frequent hospitalizations.

### Quality of life

Evaluating SF-36, all domains showed significantly lower scores compared to the normal population (Table 2).

**Table 2:** Results of short-form 36 health survey for patients with neurofibromatosis type 1 compared with German standard population.

	**NF1, Mean ±SD (*n* = 89)**	**German standard population, mean ±SD (*n* = 2,773)**	***p***
Physical component summary	48.34 ± 10.61	50.21 ± 10.24	0.045
Mental component summary	42.85 ± 7.60	51.54 ± 8.14	<0.001
Physical functioning	82.36 ± 23.25	96.61 ± 10.04	<0.001
Role: physical	70.51 ± 40.69	96.89 ± 13.88	<0.001
Bodily pain	71.54 ± 30.18	94.60 ± 14.99	<0.001
General health	61.60 ± 24.16	79.89 ± 13.66	<0.001
Vitality	54.78 ± 19.67	71.90 ± 14.31	<0.001
Social functioning	53.79 ± 10.56	94.87 ± 12.33	<0.001
Role: emotional	76.40 ± 38.99	96.89 ± 14.13	<0.001
Mental health	63.46 ± 14.51	79.16 ± 13.11	<0.001

Sex, marital status and education level did not significantly influence QoL (Table 3). However, employment status was significantly associated with better mental and physical status according to SF-36 (Table 3) while age >40 was linked to lower physical but not mental component (Table 3).

**Table 3:** Comparison of physical and mental component summary according to patients'characteristics.

	**Level of education**	**Mean ±SD**	***P* value**
Physical component summary	None	46.59 ± 8.06	0.474
	Elementary school	48.87 ± 10.26	
	Middle school	46.77 ± 11.64	
	High school	50.04 ± 11.30	
Mental component summary	None	45.55 ± 5.27	0.693
	Elementary school	42.57 ± 8.68	
	Middle school	42.13 ± 6.72	
	High school	42.85 ± 7.60	
	**Employment**	**Mean** **±SD**	***P*** **value**
Physical component summary	Employed	49.54 ± 10.17	*0.010*
	Unemployed	41.30 ± 10.77	
Mental component summary	Employed	43.62 ± 7.22	*0.028*
	Unemployed	38.40 ± 8.50	
	**Partner/marital status**	**Mean** **±SD**	***P*** **value**
Physical component summary	Single	47.84 ± 11.69	0.863
	Partnered/married	48.78 ± 9.64	
Mental component summary	Single	41.84 ± 8.74	0.375
	Partnered/married	43.76 ± 6.36	
	**Age (years)**	**Mean** **±SD**	***P*** **value**
Physical component summary	≤ 40	50.08 ± 11.38	*0.027*
	> 40	46.47 ± 9.49	
Mental component summary	≤ 40	43.53 ± 7.62	0.362
	> 40	42.13 ± 7.59	
	**Sex**	**Mean** **±SD**	***P*** **value**
Physical component summary	Male	47.00 ± 11.93	0.530
	Female	49.12 ± 9.77	
Mental component summary	Male	43.61 ± 7.88	0.316
	Female	42.41 ± 7.46	

Italic values indicate *p* < 0.05.

According to numerical pain rating scale, 7–10 were noted in 9 cases (10,1%), 5–6 in 10 patients (11.2%), 1–4 in 26 patients (29.2%) and no pain in 44 cases (49.4%). Physical component showed significant difference between different NPS grades (*P* < 0.001, Table 4) but no significant difference in mental component summery (*P* = 0.06).

**Table 4:** Comparison of physical and mental component summery between different grades of disease severity.

	**Numerical rating scale**	**Mean ±SD**	**P value**
Physical component summary	0	54.30 ± 7.21	* < 0.001*
	1–4	46.89 ± 8.70	
	5–6	41.28 ± 6.79	
	7–10	31.21 ± 8.65	
Mental component summary	0	42.25 ± 7.81	0.064
	1–4 (*n* = 26)	45.93 ± 6.16	
	5–6 (*n* = 10)	40.96 ± 8.01	
	7–10 (*n* = 9)	39.03 ± 7.91	
	**Visibility scale**	**Mean** **±SD**	**P value**
Physical component summary	Grade 1	53.30 ± 8.04	* < 0.001*
	Grade 2	47.69 ± 10.69	
	Grade 3	42.73 ± 10.71	
Mental component summary	Grade 1	42.63 ± 7.31	0.840
	Grade 2	42.83 ± 6.98	
	Grade 3	43.16 ± 7.60	
	**Disease severity**	**Mean** **±SD**	**P value**
Physical component summary	Grade 1 (*n* = 7)	54.90 ± 7.92	*0.006*
	Grade 2 (*n* = 23)	52.02 ± 10.21	
	Grade 3 (*n* = 17)	43.00 ± 10.78	
	Grade 4 (*n* = 42)	47.38 ± 10.16	
Mental component summary	Grade 1 (*n* = 7)	46.18 ± 2.96	0.678
	Grade 2 (*n* = 23)	41.04 ± 8.82	
	Grade 3 (*n* = 17)	42.92 ± 5.88	
	Grade 4 (*n* = 42)	43.27 ± 7.96	

Italic values indicate *p* < 0.05.

No significant differences were found between different severity grades and visibility grades in mental component summery (Table 4). As expected, physical components were significantly worse in more severe cases (Table 1) and in patients with higher visibility grades.

## Discussion

The QoL is one of the most relevant outcome parameters in patients with chronic diseases (11–13). Generally, all patients in our study showed reduced QoL based on SF-36. This aspect highlights the significant impact of NF1 on quality of life, which is consistent with previous studies (14, 15).

Regarding the visible aspects of this neurocutaneous disease, that contrary to previous studies, we did not observe significant differences in mental health scores across different severity groups. Chren et al. and Krueger et al. showed in contrast, that disorders that affect the skin, result in negative emotional and psychological outcomes (16, 17). Kodra et al. have found similar results in NF 1 where the changing of the appearance because of the skin abnormalities ends in an inferior QoL (7). Smith et al. reported that the female sex is especially affected by cosmetic burdens of the NF1 (18). Similarly, Hummervoll et al. noted that females had tremendously worse QoL in contrast to men (19). This is in discrepancy to our results which showed no significant difference between males and females. In a similar way to our findings, Crawford et al. haven't found gender differences nor even an influence of visibly changes of the QoL in the Australian population (20). The participants of our study may cope better with the change of the appearance or have a better body image than we have expected. Many participants cope with the visible aspects of the NF1 by concealing the skin with special clothing or avoiding activities like swimming (2). Similarly to our study, a Canadian publication also showed no significant differences in the body image scores of women compared to men (21).

Previous research has suggested visible aspects of NF1 can pose challenges in forming relationships and finding a partner is more difficult (22). We haven't found a relevant difference compared to the standard population in our study (22).

It is known that the attractiveness is positively influencing the state of employment, so it is to be expected, that the skin abnormalities of the NF1 leads to a higher number of unemployment, but in contract to former studies, the participants of our study have a normal level of employment (23, 24). The level of education is lower, which is correlating with the type of employment. Many participants in our study are manual laborers in factories or working as unskilled workers, where the visual appearance is not so important.

A recent review and meta-analysis by Crow et al. also showed that cognitive deficits in this group are widespread and significant (25). Not all areas of cognitive function are equally affected. Age, gender, education level and parental education level have no significant impact on cognitive outcomes. This underscores the need for early and continuous support of cognitive functions in patients with NF1 throughout their lifespan. Additionally, recent Finnish research indicates that NF1 is associated with lower educational attainment and a tendency to purse vocational rather than academic education. Individuals living with NF1 particularly those with cancer, developmental disorders or familial NF1 require effective student counseling and learning assistance (26).

Learning difficulties are a well-known aspect in NF1 and are often the reason for painful school experiences, including social assaults and unhappiness, leading to school refusers and a drop out of trainings (8, 22). The results of these learning difficulties are often a lack of self-confidence, missed career choices and employment opportunities. In our cohort, the level of education is lower compared to the German population (27). Especially the number of people without any graduation is higher and the number of participants having a university degree is much lower. These results are in line with former studies (28). Other studies describe that older adults (born before 1970) have worse school experiences than younger ones, this may be explained by a greater awareness of NF1 and leads to a necessity of an early support and the treatment of the learning difficulties and the lack of concentration (29).

Pain is a prevalent and significant factor affecting QoL, so we used except of the SF-36 questionnaire and the patient history, the NRS to correlate the severity of the pain with the QoL. Nearly half of the population described no pain, but the physical component showed significant difference with increasing NRS. In our study, participants predominantly reported back pain or headaches, which are typical manifestations of NF1–on the one hand attributed to the typical bodily findings in the NF1 like scoliosis and poor postures, but on the other hand it can be a sign of psychological disorders like depression and maladaptive coping strategies associated with the chronic nature of the disease (30, 31).

Brar et al. published a study in 2023 that highlights the prevalence of psychiatric comorbidities in NF patients, particularly associated with male sex and for people of color. Mood disorders and anxiety disorders were the most common, while ADHD was less prevalent than in previous studies. This further emphasizes the importance of psychological support for this patient group (32).

It's important to investigate and treat the physical restrictions in the early childhood to avoid later problems. Emphasis should be placed on implementing multidisciplinary approaches to integrate psychological therapies such as acceptance and commitment therapies (ACT), resilience and coping strategies (1, 14, 29). Furthermore, regular neuropsychological assessment with regard to visual spatial skills and attention deficits was recommended for further support and improvement of QoL in children (33). Cavallo et al. reported recently that in children population of patients with NF1 disease severity interferes with social functioning and consequently QoL (34). This may lead to stigmatization which could be less relevant in the adult population as presented in our results. The early identification of QoL in both pediatric and adult population with an early intervention and personalized treatment might improve further wellbeing of this patients cohort.

Regarding the influence of the age, many studies demonstrate a lower QoL in younger NF1 patients, a pattern observed in other chronic diseases (35, 36). Although our study did not include children, we mentioned, that the age >40 years is significantly associated with a lower physical component. Probably, possible bone abnormalities like scoliosis, plexiform neurofibromas and a higher risk of developing malignant tumors as well as associated surgeries can explain the higher physical problems of this group. Surprisingly, the mental component of QoL was not affected in this age group. Probably, the coping strategies are better and the life and family planning is completed. In addition, there are not enough investigations to report the differences between adults and children with NF1 (even separated by children and parents‘ reports) or long-term follow-ups, regarding the development of this population over the years.

A literature review was conducted by Domon-Archambault et al. on the social life, mental health and QoL of children and adolescents with NF1, as well as the psychosocial interventions aimed at this population (37). Compared to unaffected children and adolescents in the general population, pediatric patients with NF1 face a higher risk of experiencing social difficulties, mental health disorders, behavioral and emotional problems, and reduced QoL. There are not enough articles which discuss interventions specifically targeting the NF1 population to address these challenges. There is a pressing need to develop and evaluate psychosocial interventions for patients with NF1.

Our detailed analysis revealed that severity grade does not correlate with inferior mental status despite physical functioning in more severe cases. In former studies, the principal concerns of the participants were the cosmetic neurofibromas followed by learning difficulties and across all age groups and gender the fear of disease progression. Interestingly the measured severity of disease using the Huson scale did not directly correlate with individual perceptions of this disease. Some participants seem to cope better with their chronic disease and their acceptance of their body seems to be much better. Especially the impact of psychosocial factors, due to the lack of treatment methods and the limitations of medication in NF1 should be considered to have the opportunity to develop resiliency strategies (15).

Based on our finding and experience, an early investigation of NF1 children through specialized centers coupled with individualized therapies is essential for promoting optimal development and long-term wellbeing. Increasing awareness of the NF1 among healthcare providers and the general public is key to improving diagnosis, treatment and support services for affected individuals and their families.

## Study limitations

One of the primary limitations of our study is the relatively small and heterogenous cohort of participants. The limited sample size and diversity in patient characteristics may restrict the generalizability of our findings to broader populations of individuals with NF1. Consequently, our results may not fully represent the QoL experiences of all NF1 patients, and caution should be exercised when applying these findings to larger, more diverse populations.

The detailed analysis of QoL in NF1 patients is inherently complex and influenced by multiple factors that are challenging to summarize and quantify.

The Ablon scale has certain limitations as well. While Ablon's Visibility Index measures the visibility of the disease, it does not evaluate the severity of the condition, such as the necessity for surgeries, various medications, such as chemotherapeutics or other treatments.

Quality of life encompasses physical, psychological and social dimensions, each can be affected by the multifaceted nature of NF1. As a result, our study may not fully capture the nuanced interactions and variability in QoL experiences among individuals with NF1.

The multitude of factors influencing QoL in NF1, including disease severity, symptom variability, psychosocial factors and treatment interventions, pose challenges in summarizing and interpreting study outcomes. Quantifying the impact of these diverse factors on overall QoL outcomes requires comprehensive and detailed assessments, which may not have been fully achieved in our study due to limitations in data collection and analysis.

## Conclusion

The severity and visibility grade of NF1 patients may not necessarily result in poor mental health in comparison with lower grades. Employment was associated with better QoL according to our results. Based on that, it is important to support this group of patients to protect their jobs and even if the level of education was not significant for the QoL, it seems to be reasonable to support the younger patients with NF1 to minimize learning disabilities and to acquire a graduation and thereby an employment.

## Data Availability

The original contributions presented in the study are included in the article/supplementary material, further inquiries can be directed to the corresponding author.
